# Somatic Embryogenesis as a tool for forest tree improvement: a case- study in *Eucalyptus globulus*

**DOI:** 10.1186/1753-6561-5-S7-P128

**Published:** 2011-09-13

**Authors:** Gisele Andrade, Ravi Shah, Sofie Johansson, Gloria Pinto, Ulrika Egertsdotter

**Affiliations:** 1 Department of Forest Genetics and Plant Physiology, Swedish University of Agricultural Sciences, Umeå, 90187, Sweden; 2 2Centre for Environmental and Marine Studies (CESAM) & Department of Biology, University of Aveiro, 3810-193, Aveiro, Portugal

## Background

Somatic embryogenesis (SE) technology is expected to play a significant role in future forest tree improvement programs. Methods for efficient SE propagation in softwood species based on bioreactors are underway [1]. Furthermore, genetic transformation through SE is also the most promising approach to generate and propagate elite recalcitrant genotypes of forest trees [2], or clonal propagation of selected endangered high-value forest trees [3,4].

Somatic embryogenesis induction was first reported for the genus *Eucalyptus* in 1980. Mass production of SE plants however remains difficult although procedures designed for plant regeneration have been reported for *E. citriodora*, *E. grandis*, *E. tereticornis*[5]. Recent data on *E. globulus* are promising and show the potential of this technology for application in clonal forestry [6,7].

A service platform for routine transformation of forest trees has been established at the Berzelii Center at Umeå Plant Science Centre using Norway spruce as the primary model species for gene function analyses. Other target species for future studies include hardwood species such as Poplar and Eucalyptus. We present here a brief summary of a case study for introduction of a recalcitrant forest species to the platform as demonstrated by the establishment of the SE technology for *E. globulus* following protocol previously established in Portugal [6,7] and preliminary results aiming to improve the induction of SE.

## Material and methods

Half-sib seeds of *E. globulus* collected from trees in Portugal (Altri Florestal Breeding Program) were used in this study. Seeds were surface-disinfected with a mixture of 1:1 absolute ethanol: hydrogen peroxide 30% (v/v) for 15 minutes. Two sources of explants were tested: (a) cotyledons and hypocotyls isolated from 15 day-old *in vitro* seedlings germinated on standard MS medium and (b) zygotic embryos (ZE) isolated after removal of the seed coat. Briefly, using a standard protocol [7], explants were inoculated on induction medium (MS_3NAA_) consisting of MS with 3 mg/l NAA, 30 g/l sucrose, 2.5 g/l Gelrite, pH 5.8. Cultures were incubated in the dark at 24^?^C for 25 days. After this period, explants were transferred to the same MS medium without NAA (expression medium MS_WH_) under the same conditions.

To evaluate the effect of factors indicated from previous studies as essential for the process of SE induction, we then tested the effect of media composition using only ZE as explants: induction and expression using MS salts but containing other vitamins (MS, B5 and RP) and 5 days induction on MS containing 5 mg/l NAA or 2 mg/l 2,4-D and the remaining 20 days on MS_3NAA_ .

Embryogenic potential was analyzed under the microscope and the results expressed as the percentage of explants showing somatic embryos. Callus production and root formation were also scored.

## Results

Adopting a standard protocol, the source of explants affected the SE induction as previously reported for other species: no embryogenic response was observed and only callus and root formation occurred when cotyledons and/or hypocotyls was used as source of explants (data not shown); 5-20% of the explants showed formation of globular somatic embryos after transferring to expression medium within 5-8 weeks after induction when ZE was used. The present study included the same OP families previously tested in Portugal. The induction rates rated in a similar way to the previous study, confirming the importance of the genotype.

Preliminaries results from the evaluation of factors affecting SE inductions show that root and root hair formation was observed on most of the explants in all tested treatments. Adding different vitamins to the MS medium promote the same embryogenic response. When ZE were cultured for short period on medium containing 2,4-D or 5 mg/l of NAA, almost 100% of the explants showed callus formation. However, the embryogenic response was similar to the control treatment indicating that concentration/induction period should be adjusted and/or other PGRs combinations tested in the future.

Individual or small clusters of globular embryos were isolated and transferred to MS_3NAA_ for capture of embryogenic cultures. The most developed somatic embryos isolated displaying a shoot and tap root were transferred to MS_WH_ for plant conversion.

## Conclusion

The data reported here show the reproducibility of the previously published protocol using different genotypes of *E.globulus*. The results also indicate the importance of genotype over culture condition for the induction success. New experiments are in progress aiming to increase the induction of SE and establish an efficient protocol that can form the basis for genetic transformation of hardwood SE cultures.
